# Subcellular spatio-temporal intravital kinetics of aflatoxin B_1_ and ochratoxin A in liver and kidney

**DOI:** 10.1007/s00204-021-03073-5

**Published:** 2021-05-18

**Authors:** Ahmed Ghallab, Reham Hassan, Maiju Myllys, Wiebke Albrecht, Adrian Friebel, Stefan Hoehme, Ute Hofmann, Abdel-latif Seddek, Albert Braeuning, Lars Kuepfer, Benedikt Cramer, Hans-Ulrich Humpf, Peter Boor, Gisela H. Degen, Jan G. Hengstler

**Affiliations:** 1 Leibniz Research Centre for Working Environment and Human Factors, Technical University Dortmund, Ardeystr. 67, 44139 Dortmund, Germany; 2 Department of Forensic Medicine and Toxicology, Faculty of Veterinary Medicine, South Valley University, Qena, 83523 Egypt; 3 Institute of Computer Science, Saxonian Incubator for Clinical Research (SIKT), University of Leipzig, Haertelstraße 16-18, 04107 Leipzig, Germany; 4 Dr. Margarete Fischer-Bosch Institute of Clinical Pharmacology, Auerbachstr. 112, 70376 Stuttgart, Germany; 5 Department of Food Safety, German Federal Institute for Risk Assessment, Max-Dohrn-Str. 8-10, 10589 Berlin, Germany; 6 Institute of Systems Medicine with Focus on Organ Interactions, University Hospital RWTH Aachen, Pauwelsstr. 19, 52074 Aachen, Germany; 7 Institute of Food Chemistry, Westfälische Wilhelms-Universität Münster, Corrensstr. 45, 48149 Münster, Germany; 8 Institute of Pathology and Department of Nephrology, University Hospital RWTH Aachen, Pauwelsstr. 30, 52074 Aachen, Germany

**Keywords:** In vivo imaging, Mycotoxins, Pharmacokinetics, Two-photon

## Abstract

Local accumulation of xenobiotics in human and animal tissues may cause adverse effects. Large differences in their concentrations may exist between individual cell types, often due to the expression of specific uptake and export carriers. Here we established a two-photon microscopy-based technique for spatio-temporal detection of the distribution of mycotoxins in intact kidneys and livers of anesthetized mice with subcellular resolution. The mycotoxins ochratoxin A (OTA, 10 mg/kg b.w.) and aflatoxin B_1_ (AFB_1_, 1.5 mg/kg b.w.), which both show blue auto-fluorescence, were analyzed after intravenous bolus injections. Within seconds after administration, OTA was filtered by glomeruli, and enriched in distal tubular epithelial cells (dTEC). A striking feature of AFB_1_ toxicokinetics was its very rapid uptake from sinusoidal blood into hepatocytes (*t*_1/2_ ~ 4 min) and excretion into bile canaliculi. Interestingly, AFB_1_ was enriched in the nuclei of hepatocytes with zonal differences in clearance. In the cytoplasm of pericentral hepatocytes, the half-life (*t*_1/2_~ 63 min) was much longer compared to periportal hepatocytes of the same lobules (*t*_1/2_ ~ 9 min). In addition, nuclear AFB_1_ from periportal hepatocytes cleared faster compared to the pericentral region. These local differences in AFB_1_ clearance may be due to the pericentral expression of cytochrome P450 enzymes that activate AFB_1_ to protein- and DNA-binding metabolites. In conclusion, the present study shows that large spatio-temporal concentration differences exist within the same tissues and its analysis may provide valuable additional information to conventional toxicokinetic studies.

**Supplementary Information:**

The online version contains supplementary material available at 10.1007/s00204-021-03073-5.

## Introduction

Concentrations of toxic substances may vary considerably within a tissue. These spatial differences can be caused by active transport mechanisms or physicochemical properties. Tissues usually consist of different cell types with distinct abilities to enrich specific substances. For instance, particles of > 100 nm or LPS are preferentially taken up by tissue macrophages of the liver and much less by hepatocytes (Koppert et al. 2018; Reif et al. 2017). Vice versa, substrates of the hepatocyte uptake carriers NTCP or OATPs may be enriched in hepatocytes but not in macrophages (Jansen et al. 2017; Reif et al. 2017). Differences may also exist between individual cells of the same type, such as hepatocytes in the central and periportal regions of liver lobules. Moreover, high local concentrations of substances may be generated by active transport processes into space-restricted tissue compartments, for example when hepatocytes secrete bile acids or xenobiotics into narrow bile canaliculi (Ghallab et al. 2019a). Locally increased concentrations may lead to toxicity, although additional parameters, such as the cell-type-specific toxifying or detoxifying metabolic capacity, play a critical role (Albrecht et al. 2019; Leist et al. 2017).

Time concentration profiles of chemicals in blood or tissues are often described by physiologically-based pharmacokinetic (PBPK) models (Schenk et al. 2017). However, even relatively advanced PBPK models consider individual organs as single compartments (Thiel et al. 2015) or at most differentiate a limited number of compartments within one organ (Bartl et al. 2015; Ghallab et al. 2016; Schliess et al. 2014). Relatively little is known about local concentrations in sub-compartments of tissues (Reif et al. 2017; Schneider et al. 2021; Schuran et al. 2021). Experimentally, local distributions of test compounds in tissues can be analyzed by autoradiography (Groothuis et al. 1982). However, this technique does not allow the analysis of temporal changes in the same tissue. Similarly, matrix-assisted laser desorption/ionization mass spectrometry imaging (MALDI MSI) cannot be performed in living tissue (Sezgin et al. 2018). These limitations may be overcome by intravital two-photon imaging (Ghallab et al. 2019a; Koppert et al. 2018; Koeppert et al. 2021). This technique offers the possibility to quantitatively study fast processes in the millisecond range but also kinetics that requires long-term analysis of several hours (Reif et al. 2017). The spatial resolution of ~ 200 nm allows the analysis of subcellular structures, such as vesicles or mitochondria. Moreover, differentiation of nuclei and cytoplasm is easily possible. Importantly, the technique can be used to study processes in intact organs of living mice (Ghallab et al. 2019a; Reif et al. 2017; Vartak et al. 2021). Excitation is achieved by an infrared laser with two photons, each transferring half of the energy required for excitation, and occurs only at the focal plane (Benninger and Piston 2013). Thus, imaging is possible with reduced phototoxicity and photo-bleaching in comparison to confocal microscopy (Reif et al. 2017).

In the present study, we evaluated the possibilities and limitations of two-photon-based intravital imaging to study the spatio-temporal kinetics of two mycotoxins in intact livers and kidneys of mice. For this purpose, we used the mycotoxins ochratoxin A (OTA) and aflatoxin B_1_ (AFB_1_), since the toxicity, pharmacokinetics, and physicochemical properties of these compounds are well-established (Table 1). OTA causes nephrotoxicity and renal tumors in several animal species (EFSA 2020a; Mally and Dekant 2009; O’Brien and Dietrich 2005). Due to its high plasma protein binding (> 99%), OTA has a very long half-life in the blood (Ringot et al. 2006). In contrast, AFB_1_, one of the strongest liver carcinogens (IARC 2012), has a relatively short half-life in the blood (Table 1). Both AFB_1_ and OTA show a blue auto-fluorescence, which enables their label-free detection in vivo. Here, we report that both compounds can be detected in intact organs of living mice with a remarkably high spatio-temporal resolution. AFB_1_ and OTA show tissue and cell-type specificities with distinct rates of accumulation and clearance from subcellular compartments. Several of the here observed aspects of spatio-temporal kinetics show a much higher intra-tissue heterogeneity than previously expected, such as the much slower clearance of AFB_1_-associated fluorescence from pericentral than periportal hepatocytes, the remarkable speed of AFB_1_ transport from the sinusoidal blood to bile canaliculi, and a stronger accumulation of OTA in distal compared to proximal renal tubular epithelial cells. The here described technique opens new perspectives to determine pharmacokinetics in living tissues, including the possibility to analyze large tissue regions for several hours or to image subcellular domains with fast sequences in the millisecond range.

**Table 1:** Overview on physicochemical, kinetic and toxicological properties of aflatoxin B_1_ and ochratoxin A, the tested mycotoxins

Compound	Aflatoxin B_1_ (AFB_1_)	Ochratoxin A (OTA)
Structure		
CAS-number	1162–65–8	303–47–9
Molecular formula	C_17_H_12_O_6_	C_20_H_18_ClNO_6_
Molecular mass (g/mol)	312.3	403.8
Log *P*	1.23	4.37
pKa	n.a	4.3 and 7.2 (weak organic acid)
Albumin (HSA) binding *K*	~ 10^4^ (log *K* 4.65) (Poor et al. 2017)	~ 10^7^ (log *K* 7.0–7.6) (Poor et al. 2013)
Acute toxicity (LD_50_) in mice	C57Bl/6 (i.p.) > 15 mg/kg b.w. (O’Brien et al. 1983) > 60 mg/kg b.w. (Almeida et al. 1996)	Range (i.v.) 25.7–33.8 mg/kg b.w Range (p.o.) 46–58.3 mg/kg b.w. (IARC 2002)
Half-life in blood (mice, humans)	Mice: *t*_1/2_ 12.9 min (Wong and Hsieh 1980) after i.v. injection Human: *t*_1/2 alpha_ 2.9 h and *t*_1/2 beta_ 64.6 h (Jubert et al. 2009) after p.o	Mice: *t*_1/2_ 40 h (p.o.) Human: *t*_1/2_ 35.5 days (O’Brien and Dietrich 2005; Ringot et al. 2006)
Metabolism (in rodents and humans)	Bio-activation to 8,9–AFB_1_–epoxides; detoxification of AFB_1_–epoxide by GSTs; hydroxylated metabolites such as AFM1, AFQ1, AFP1, (Deng et al. 2018; Guengerich et al. 1998)	OTA, the toxic principle, undergoes hydrolysis to OT-alpha in the GI-tract; minor metabolites are 4R/4S- and 10-hydroxy-OTA (less toxic than OTA); glucuronidation of OTA and OT-alpha as well as GSH adduct formation (Ringot et al. 2006; Duarte et al. 2011; Sueck et al. 2020)
Genotoxicity and mode of action	Formation of *exo*–AFB_1_–8,9–epoxide leads to induction of pre-mutagenic DNA adducts (AFB_1_–N7–Gua and AFB_1_–FAPY adducts) which produce mainly G-to-T transversions and mutations. Convincing evidence for a mutagenic mode of action (EFSA 2020a; IARC 2012)	OTA-induced genetic damage (strand breaks, micronuclei) observed in vitro independent of metabolic activation; some genotoxic effects may be secondary to oxidative stress. OTA is a weak mutagen in vivo. Formation of specific OTA–DNA adducts still highly controversial
Sub-chronic/chronic toxicity main target organ	Liver: dose- and time-dependent histological and biochemical changes in rodents, with male F344 rats being most sensitive. Induction of hepato-cellular carcinomas in various species, including humans (IARC 2012) Infant mice or GSTA3-KO mice far more susceptible than adult or wild-type mice (Crawford et al. 2017; Vesselinovitch et al. 1972)	Kidney: dose- and time-dependent induction of nephrotoxicity in all mammalian species tested, including mice, rats, dogs and pigs, with marked differences in sensitivity to OTA toxicity between sex and species (EFSA 2020b; Mally and Dekant 2009)
Cell type-specific toxicity	Repeated doses of 0.75 and 1.5 mg/kg b.w. caused hepatocellular necrosis in mice (Jha et al. 2013)	Histopathological changes of the S3 segment of proximal tubules in mice (Bondy et al. 2015)

## Materials and methods

### Mice and chemicals

Male C57Bl6/N 6–8 week-old (Janvier Labs, France) or mT/mG reporter (Muzumdar et al. 2007; The Jackson Laboratory, ME, USA) mice were used in this study. The mice were housed under 12 h light/dark cycles at controlled ambient temperature of 25 °C, and were fed ad libitum with standard diet (Ssniff, Soest, Germany) and had free access to water. The experiments were approved by the local authorities. Aflatoxin B_1_ (AFB_1_) and ochratoxin A (OTA) were purchased from Sigma-Aldrich, Darmstadt, Germany (Cat. No. A6636 and O1877, respectively). OTA was also isolated from fungal cultures as described by Bittner et al. (2013) and provided by the Institute of Food Chemistry, WWU Münster, Germany. Tetramethylrhodamine–ethyl ester (TMRE) was purchased from Thermo Scientific, MA, USA (Cat. No. T669). Cholyl–lysyl–fluorescein (CLF; a bile acid analog) was obtained from BD Biosciences, California, USA (Cat. No. 451041).

### Two-photon based intravital imaging

Functional intravital imaging of OTA and AFB_1_ uptake and clearance by the liver and the kidneys was performed using an inverted two-photon microscope LSM MP7 (Zeiss, Jena, Germany) with an LD C-Apochromat 40×/1.1 water immersion objective, as previously described (Ghallab et al. 2019a; Koppert et al. 2018; Koeppert et al. 2021; Reif et al. 2017). First, anesthesia was induced in mice by intraperitoneal injection of ketamine (100 mg/kg b.w.), xylazine (10 mg/kg b.w.), acepromazine (1.7 mg/kg b.w.), and buprenorphine (0.08 mg/kg b.w.). In case of using wild-type mice, a bolus of TMRE (Table 2) was administered in the tail vein in order to visualize tissue morphology. To allow the administration of OTA and AFB_1_ while recording, a mouse catheter (SAI-infusion, IL, USA) was fixed in the tail vein.

**Table 2:** Fluorescent markers and reporter mice used in the study

Fluorescent marker/reporter	Marker for	Dose (mg/kg b.w.)	Vehicle	Two-photon excitation range (nm)
mT/mG mouse	Tissue morphology	–	–	720–800
TMRE	Tissue morphology	0.96	Methanol: PBS (1:1)	740–820
Ochratoxin A	Ochratoxin A	10	0.1 M NaHCO_3_	740–780
Aflatoxin B_1_	Aflatoxin B_1_	1.5	DMSO: PBS (1:1)	740–780
Cholyl–lysyl–fluorescein	Bile acid analog	1	PBS	740–820

### Preparation of the liver

To prepare the liver for intravital imaging, a midline incision was made in the abdominal wall caudal to the sternum. The influence of breathing on liver movement was minimized by cutting the coronary ligament which connects the liver to the diaphragm. The left liver lobe was exposed by gently pushing the mouse back.

### Preparation of the kidney

To expose the kidney for intravital imaging, the mouse was placed on its right side and an incision was made in the abdominal wall at the left flank region above the position of the kidney. The convex surface of the left kidney was then exposed by gently pushing the abdominal viscera.

The exposed liver lobe/kidney was placed onto a cover slip (0.17 mm-thick; Logitech, Glasgow, UK) fitted in a custom-made image platform. Finally, the mouse with its exposed liver lobe/kidney was moved to the microscope stage, at controlled ambient temperature of 36 °C. The exposed liver lobe/kidney was covered with a saline-soaked piece of gauze to avoid tissue dehydration. The quality of surgical preparation of the exposed liver lobe or kidney was insured by the regular blood flow.

To record baseline auto-fluorescence, image acquisition was performed for few seconds prior to bolus administration of OTA (10 mg/kg b.w.) or AFB_1_ (1.5 mg/kg b.w.) via the tail vein catheter (Table 2). At the end of AFB_1_ imaging in the liver tissue, a bolus of the green-fluorescent bile acid analog CLF (Table 2) was administered via the tail vein catheter to confirm the position of bile canaliculi. When recording was extended longer than one hour, anesthesia was maintained by exposing the mouse to isoflurane (0.5–1%) using an isoflurane inhaler. At least three mice were analyzed for each of the experimental scenarios shown in the “Results” section.

### Image analysis

Quantification of mean fluorescence intensity of OTA and AFB1 in various compartments of the liver lobule (sinusoids, hepatocyte cytoplasm and nuclei, and bile canaliculi) and the nephron (glomerular capillaries, Bowman’s space, proximal and distal tubules) was done in specified regions of interest using ZEN software (Zeiss, Jena, Germany) as previously described (Koeppert et al. 2021; Reif et al. 2017).

### Pharmacokinetic analysis

The decay in the intensity signal was first filtered by applying moving average smoothing. A linear decay was then assumed at the initial clearance phase and the half-life was calculated from the gradient (Twitchett and Grimsey 2012).

## Results

### The rapid uptake of OTA into tubular epithelial cells

**Fig. 1:**
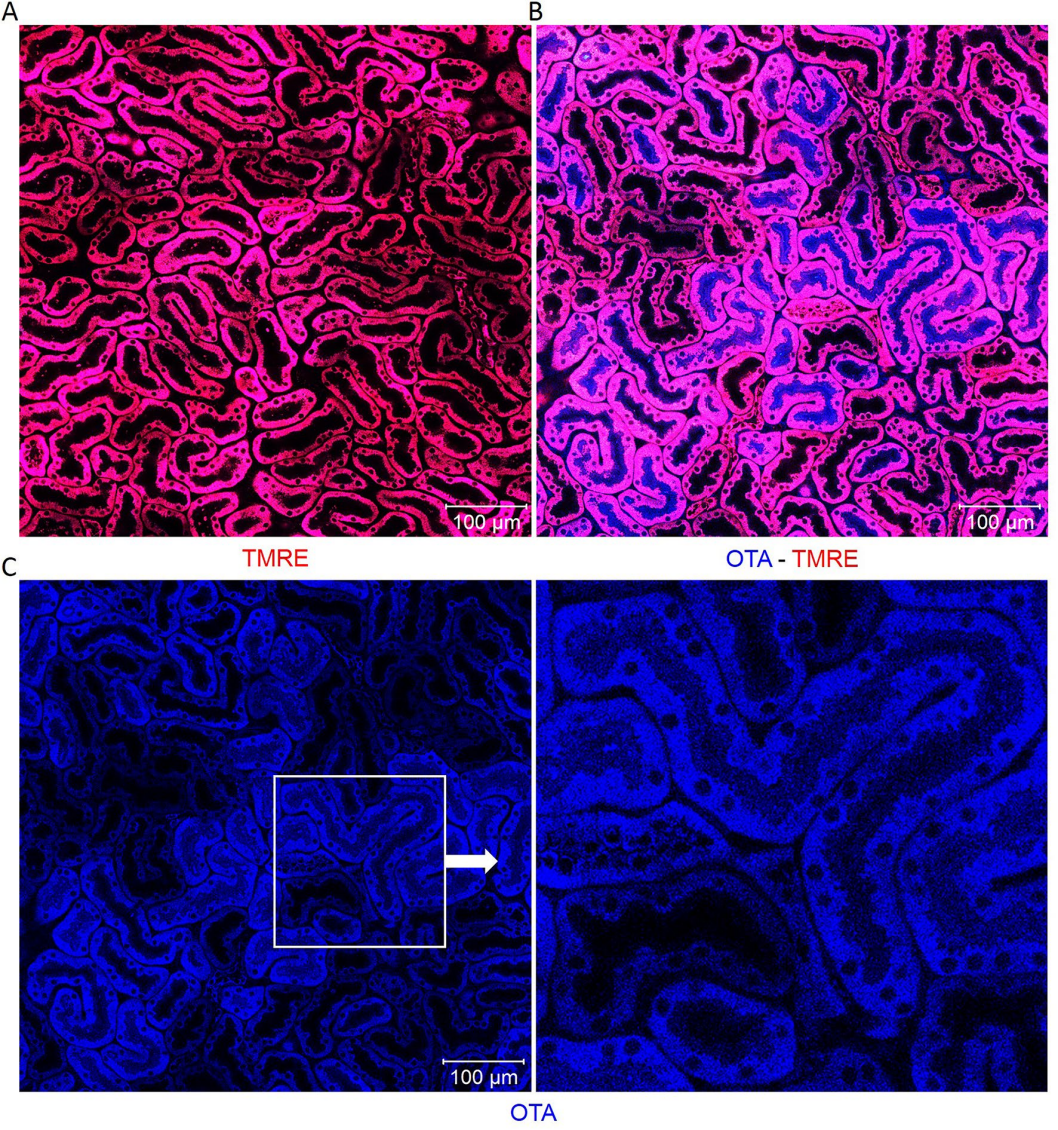
Intravital imaging of ochratoxin A (OTA) uptake by renal tubular epithelial cells. Mitochondrial membrane potential is visualized by the vital dye TMRE (red). OTA is identified by its blue auto-fluorescence. **a** Renal tubules before injection of OTA. **b** Zonated enrichment of OTA in tubular epithelial cells within a minute after intravenous bolus injection of 10 mg/kg b.w. **c** The same image seen in **b** but without the red channel (TMRE) confirming enrichment of OTA within certain tubular epithelial cells. Scale bars: 100 µm (color figure online)

Intravital two-photon imaging was performed to study the spatio-temporal kinetics of OTA in the kidney tissue of mice. The vital dye tetramethylrhodamine–ethyl ester (TMRE) was used to visualize the mitochondrial membrane potential of the tubular epithelial cells. This method allows the differentiation of two types of tubular epithelial cells, showing higher or lower mitochondrial activity as evidenced by more or less intensive TMRE-associated red fluorescence at the basolateral membrane of the tubular epithelial cells (Fig. 1a). Since OTA shows blue auto-fluorescence, its appearance in the tissue could be monitored by the two-photon microscope. For this purpose, a bolus of 10 mg/kg b.w. OTA was administered into the tail vein. Strikingly, OTA showed a clear preference for enrichment in certain tubular epithelial cells within one minute after injection (Fig. 1b, c).

**Fig. 2:**
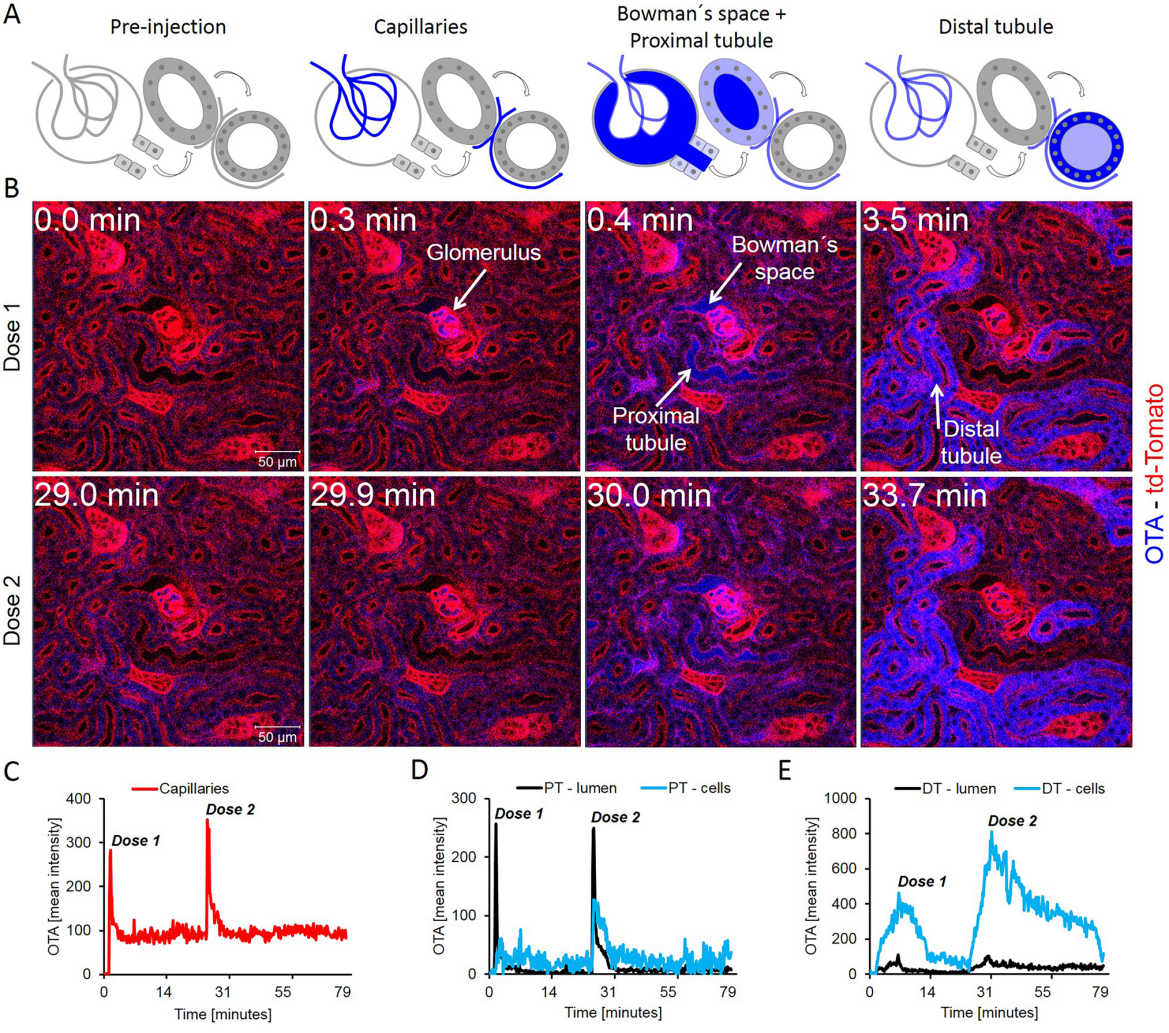
Enrichment of OTA in the distal renal tubular epithelial cells. **a** Schedule of the anatomical compartments that can be differentiated by intravital imaging. **b** Stills from intravital videos after two intravenous doses of 10 mg/kg b.w. OTA given to the same mouse at minutes 0 and 29. Red: td-Tomato; blue: OTA; scale bars: 50 µm. **c** Quantification of the OTA signal in glomerular capillaries. **d** Quantification of the OTA signal in the lumen and in the epithelial cells of proximal tubules (PT). **e** OTA signal in the lumen and in the epithelial cells of the distal tubules (DT). The figure corresponds to supplemental video 1 (color figure online)

To gain insight into the spatio-temporal kinetics of OTA in the nephron, time-lapse videos were recorded after bolus intravenous injections of OTA in mT/mG (td-Tomato) mice; a reporter mouse expressing red fluorescence in all cell membranes (Muzumdar et al. 2007) (Fig. 2, Supplemental Video 1). Within seconds after injection, OTA appeared in the glomerular capillaries, shortly thereafter (0.4 min) it was filtered to the Bowman’s space and reached the lumen of the proximal tubules, and then enriched in the epithelial cells of the distal tubules and reached peak accumulation within 4 min (Fig. 2a, b). Interestingly, the proximal tubular epithelial cells—directly adjacent to the glomerulus—enriched OTA to a much lower degree (Fig. 2a, b; Supplemental Video 1). Injection of a second bolus of 10 mg/kg b.w. OTA into the same mouse demonstrated a qualitatively similar series of events (Fig. 2b, lower panel; Supplemental Video 1). Quantification of OTA-associated blue fluorescence in specific compartments demonstrated the sharp increase in the glomerular capillaries, followed by a rapid decrease within seconds (Fig. 2c); however, the signal of OTA in the glomerular capillaries did not return to the basal level observed before injection, and the remaining signal after the first few seconds was constant during the entire recorded period (Fig. 2c; Table 3). The transient increase of OTA fluorescence in the lumen of proximal tubules followed the kinetics of the glomerular capillaries with only a slight time lag (Fig. 2d). In the cytoplasm of proximal tubular epithelial cells, a slight transient increase of OTA-associated blue fluorescence was observed immediately after the occurrence of the signal in the tubular lumen. Interestingly, a fundamentally different scenario was obtained in the distal tubules (Fig. 2e). Although the signal in the tubular lumen was weaker, the corresponding intracellular increase in distal tubular epithelial cells was much higher compared to the proximal tubular epithelial cells (Fig. 2e). Injection of a second dose of 10 mg/kg b.w OTA resulted in qualitatively similar observations but with major quantitative differences. The most striking difference compared to the first injection was the stronger increase in the distal tubular epithelial cells and the delayed clearance from these cells (Fig. 2b, e).

**Table 3:** Half-lives and t-max of ochratoxin A and aflatoxin B_1_ in various compartments of the kidney nephron and the liver lobule

Kidney						
Parameter	Mycotoxin	Capillaries	pTEC-lumen	pTEC-cells	dTEC -lumen	dTEC-cells
*t*_max_ (min)	OTA (10 mg/kg)	> 0.3	> 0.3	ND	ND	8.4
	AFB_1_ (1.5 mg/kg)	ND	ND	> 9.2	> 1.2	> 1.4
*t*_1/2_ (min)	OTA (10 mg/kg)	ND	0.4	2.8	2	2.3
	AFB_1_ (1.5 mg/kg)	0.5	0.5	0.4	1	1

Liver													
Parameter	Mycotoxin	Sinusoids	Cytoplasm periportal			Cytoplasm pericentral			Nuclei periportal		Nuclei pericentral		Canaliculi
*t*_max_ (min)	OTA (10 mg/kg)	1.6	9.3						1.4				3
	AFB_1_ (1.5 mg/kg)	1.3	1.9			6.9			2.2		8.2		1.2
*t*_1/2_ (min)	OTA (10 mg/kg)	ND	ND						ND				18
	AFB_1_ (1.5 mg/kg)	4	9				63		> 5			> 14	48

*ND* not-detectable

### Biliary excretion of OTA

**Fig. 3:**
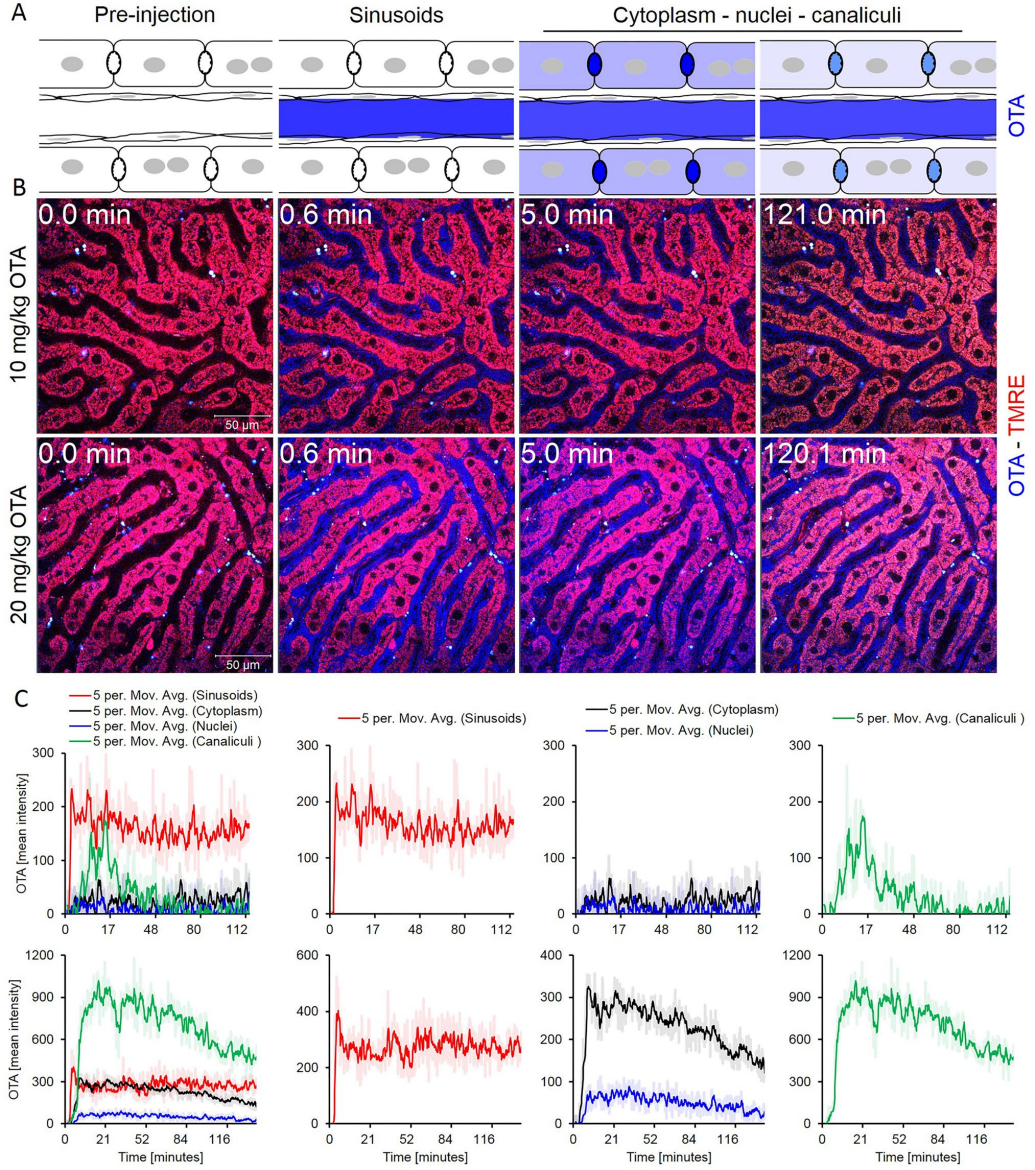
Uptake of ochratoxin A (OTA) into hepatocytes and secretion into bile canaliculi. **a** Schedule of anatomical compartments passed by OTA. **b** Stills from intravital videos after intravenous bolus injections of 10 and 20 mg/kg b.w. OTA. Red: TMRE; blue: OTA; scale bars: 50 µm. **c** Quantification of the OTA-associated blue signal in liver sinusoids, the cytoplasm of hepatocytes, nuclei of hepatocytes and in bile canaliculi after intravenous bolus injections of 10 (upper panel) or 20 (lower panel) mg/kg b.w. OTA. The figure corresponds to supplemental videos 2 and 3 (color figure online)

The same intravital imaging conditions as described above for the kidney were applied for the liver, to allow a comparison of both organs (Fig. 3a, b). After a bolus intravenous injection of OTA (10 mg/kg b.w.), a rapid increase in blue fluorescence was observed in the blood of the liver sinusoids, followed by a plateau (Fig. 3b, c; Supplemental video 2). Only a very weak increase of OTA-associated blue fluorescence was detectable in the adjacent hepatocytes and their nuclei (Fig. 3c, Supplemental Video 2). Nevertheless, a transient increase in blue fluorescence occurred in the bile canaliculi (Fig. 3c), demonstrating that OTA is enriched in this compartment by secretion from hepatocytes.

Next, a higher dose of OTA (20 mg/kg b.w.) was intravenously injected as a bolus. A correspondingly higher OTA-associated blue signal was detected in the sinusoids, also followed by a plateau (Fig. 3c, Supplemental Video 3). Interestingly, a clear increase was detected in the cytoplasm of the hepatocytes, followed by a relatively slow decrease. The kinetics in the bile canaliculi were similar to those in the cytoplasm of hepatocytes with only a very short time lag; however, the intensity of fluorescence was much higher compared to the cytoplasm of hepatocytes. The OTA-associated signal increased also in the nuclei of hepatocytes but to a much lower degree compared to the cytoplasm. Comparing the intensity of the OTA-associated signal after injection of 10 mg/kg b.w. in hepatocytes (Fig. 3c) and in distal tubular epithelial cells (Fig. 2e) demonstrates an approximately tenfold higher signal in the kidney cells.

### Transient enrichment of AFB_1_ in hepatocytes and their nuclei

**Fig. 4:**
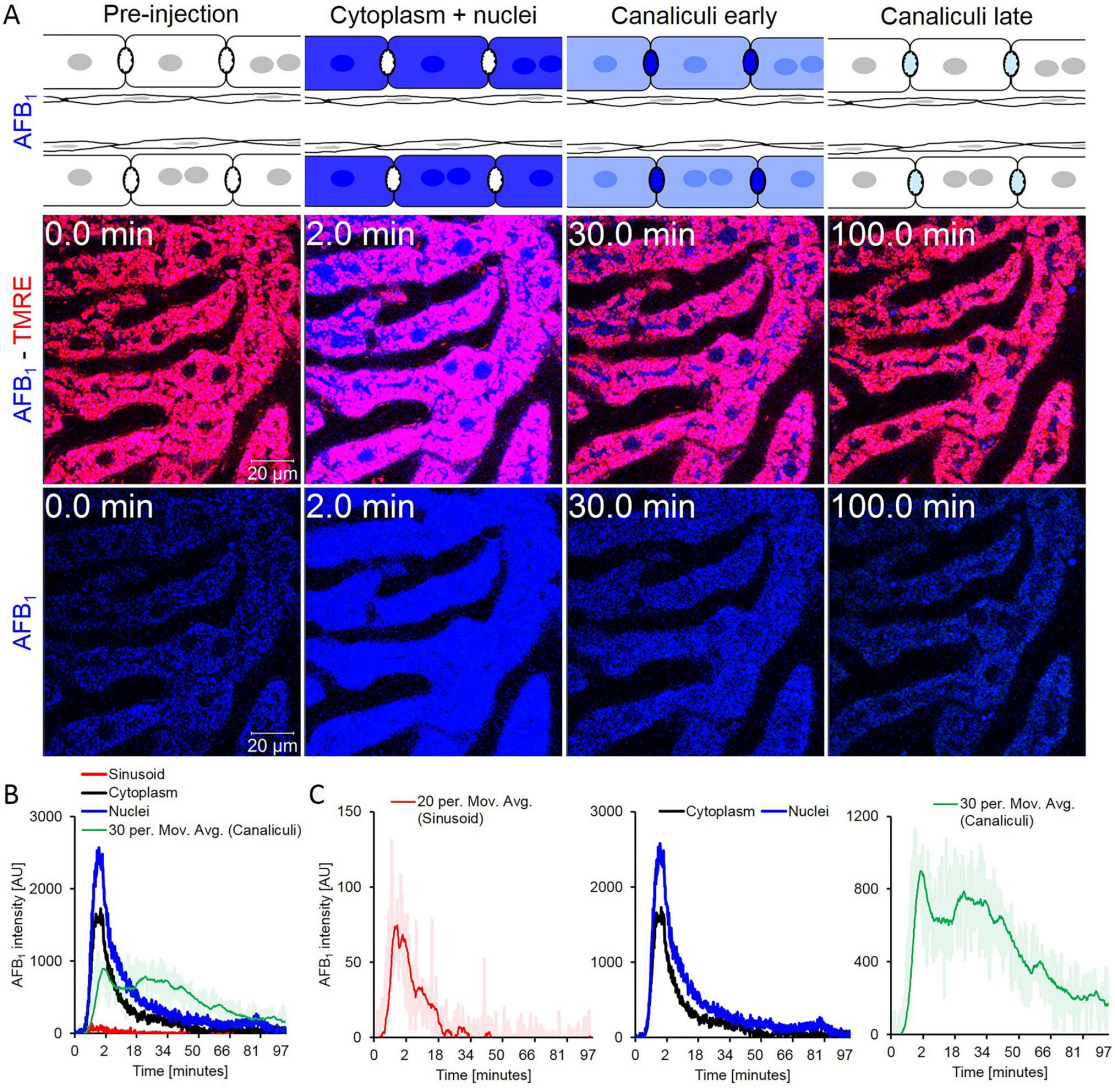
Uptake of AFB_1_ from sinusoidal blood into hepatocytes and secretion into bile canaliculi. **a** Schedule of anatomical sites passed by AFB_1_ and stills from videos after intravenous bolus injection of 1.5 mg/kg b.w. AFB_1_. Red: TMRE; blue: AFB_1_; scale bars: 20 µm. **b** Quantification of the AFB_1_-associated blue signal in the liver sinusoids, cytoplasm of hepatocytes, nuclei of hepatocytes, and bile canaliculi. **c** Quantifications shown separately for sinusoids, hepatocytes (cytoplasm and nuclei) and bile canaliculi. The figure corresponds to supplemental videos 4A and B (color figure online)

To compare the spatio-temporal kinetics of the kidney-targeted toxin OTA to the hepatotoxin AFB_1_, we performed a similar set of experiments after intravenous bolus injections of 1.5 mg/kg b.w. AFB_1_. Within only one minute after injection, AFB_1_ was taken up into the cytoplasm and nuclei of hepatocytes followed by secretion into the bile canaliculi with only a delay in the range of a few seconds (Fig. 4a, Supplemental Video 4). Quantification of the AFB_1_-associated blue signal demonstrated the increase in sinusoidal blood immediately after injection followed by a very rapid decrease within only 2–3 min (Fig. 4b, c). The AFB_1_ signal increased strongly in the cytoplasm of the periportal hepatocytes between 1 and 5 min after injection, followed by a decrease with a half-life of 9 min (Fig. 4c). Interestingly, the blue signal in the nuclei was even higher than in the cytoplasm (Fig. 4c). Regarding the extent of nuclear enrichment, AFB_1_ differed considerably from OTA, since the latter showed much stronger signals in the cytoplasm than in the nuclei (Fig. 3c). AFB_1_ was secreted into the canaliculi from where it cleared with a half-life of 48 min (Table 3).

### Longer half-lives of AFB_1_ in pericentral than periportal hepatocytes

**Fig. 5:**
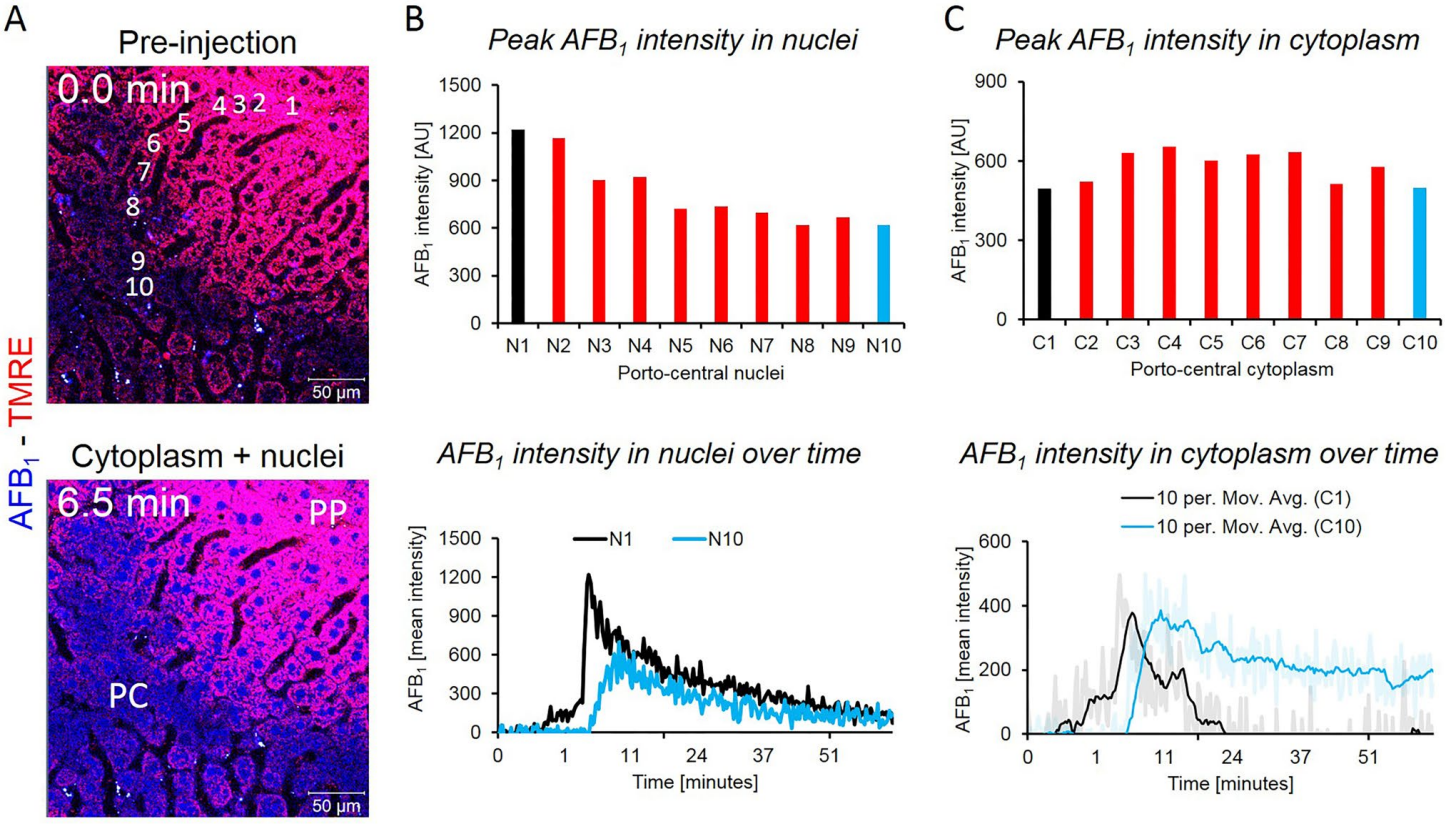
Zonation of AFB_1_ toxicokinetics. **a** Stills from a video before and 6.5 min after a bolus intravenous injection of 1.5 mg/kg b.w. AFB_1_. High intensity of the TMRE signal (red) indicates the periportal (PP), low intensity indicates the pericentral (PC) lobular region. Hepatocytes from the most PP to the PC region were numbered from 1 to 10. Red: TMRE; blue: AFB_1_; scale bars: 50 µm. **b** Upper panel: peak intensity of AFB_1_ in the nuclei of hepatocytes no. 1–10. Lower panel: time-course of the AFB_1_-associated signal in the most periportal (N1) and the most pericentral hepatocyte (N10). **c** AFB_1_ signal in the cytoplasm of hepatocytes, corresponding to the analyses shown in panel **b**. The figure corresponds to supplemental video 5 (color figure online)

Next, we studied the lobular zonation of AFB_1_ kinetics, since the bio-activating cytochrome P450 enzymes are expressed in the pericentral region of liver lobules (Gebhardt 1992; Ghallab et al. 2019b). A recently established technique to differentiate the pericentral and periportal lobular zone was applied that is based on intensity analysis of TMRE (Reif et al. 2017). Upon tail vein injection, TMRE is taken up more intensively by periportal than by pericentral hepatocytes. We positioned the imaging plane parallel to the hepatocyte sheets and numbered the hepatocytes from the most periportal position (no. 1) towards the pericentral region (no. 10) (Fig. 5a, Supplemental Video 5). The stills in Fig. 5a show a lobule before and 6.5 min after injection of AFB_1_. For each hepatocyte (no. 1–10), the AFB_1_-associated nuclear signal was analyzed time-dependently (Fig. 5b) and the peak intensity was determined. The peak of nuclear AFB_1_ intensity was higher in periportal (no. 1) than in pericentral (no. 10) nuclei. However, the half-life in the pericentral nuclei (> 14 min) determined after normalization to the peak intensity was longer compared to that in the periportal nuclei (> 5 min). The same analysis was performed for cytoplasmic AFB_1_-associated fluorescence (Fig. 5c). No association between the lobular zone and cytoplasmic AFB_1_ peak intensity was observed (Fig. 5c). However, similar to nuclear intensity, cytoplasmic intensity had a longer half-life in pericentral (*t*_1/2_ ~ 63 min) than in periportal (*t*_1/2_ ~ 9 min) hepatocytes (Table 3).

### Weak uptake of AFB_1_ by renal tubular epithelial cells

**Fig. 6:**
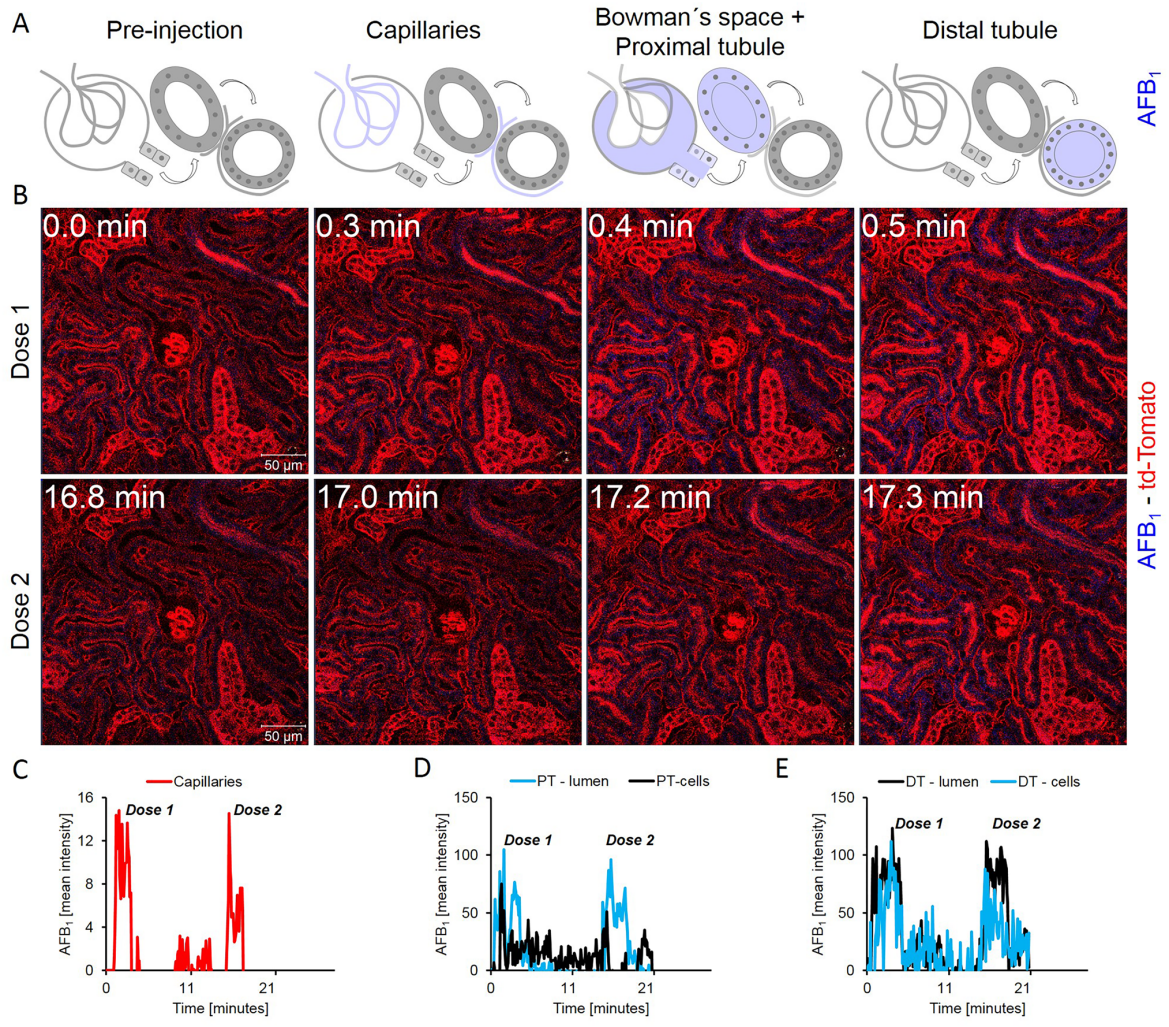
Toxicokinetics of AFB_1_ in the kidney. **a** Schedule of anatomical sites. **b** Stills from a video after bolus intravenous injections of two subsequent doses of 1.5 mg/kg b.w. AFB_1_. Red: td-Tomato; blue: AFB_1_; scale bars: 50 µm. Quantification of the AFB_1_-associated blue signal in glomerular capillaries **c**, lumen and cytoplasm of tubular epithelial cells of proximal tubules (PT; **d**) and distal tubules (DT; **e**). The figure corresponds to supplemental videos 6 (color figure online)

Similar to OTA (Fig. 2), also AFB_1_ was analyzed in the kidney (Fig. 6a, b; Supplemental Video 6). Compared to OTA, the AFB_1_-associated signal in tubular epithelial cells was very weak. A transient increase in the glomerular capillaries was detectable after a bolus intravenous injection of 1.5 mg/kg b.w. AFB_1_ (Fig. 6c). AFB_1_-associated fluorescence was also transiently detected in the lumen and the epithelial cells of the proximal tubules (Fig. 6d). Similarly, a slight transient increase of AFB_1_ signal was also detected in the lumina and epithelial cells of the distal tubules (Fig. 6e).

## Discussion

Two-photon-based intravital imaging of OTA and AFB_1_ revealed complex spatio-temporal changes of tissue concentrations in the kidney and the liver. OTA is known to have a very high affinity to albumin, and at least 99% of the OTA circulating in the blood is bound to plasma proteins (Duarte et al. 2011; Mally and Dekant 2009; Sueck et al. 2018). Together with its poor metabolism, this results in a very long plasma half-life of 40–50 h in mice after oral dosing (Table 1; Ringot et al. 2006). It should, however, be considered that OTA was injected into the tail vein in 0.1 M NaHCO_3_ (without proteins) in a relatively large volume of 2 µL/g b.w. Thus, it is possible that in the first few seconds after i.v. injection a higher fraction of non-protein bound OTA reaches the glomerular capillaries, and therefore is filtered, resulting in transiently increased concentration in the lumen of proximal and to a lower degree also in distal tubules. In contrast, in the steady state after filtration of the free fraction, a plateau of OTA concentration above pre-injection levels is established in the glomerular capillaries, probably because the now predominantly protein-bound OTA does not, or only very slowly, pass the glomerular barrier. In the period when OTA reaches a plateau (above baseline levels) in the glomerular capillaries, the OTA-associated signal in the lumen of proximal and distal tubules returns to baseline levels.

In sub-chronic studies with rodents, OTA-induced nephrotoxicity is characterized by histopathological changes (disorganization of tubule morphology, apoptosis, aberrant mitotic figures, and karyomegaly) affecting proximal tubular epithelial cells in the cortex and outer medulla (Bondy et al. 2015; Mally et al. 2005; Rached et al. 2007). This may appear surprising since we observed uptake of OTA from the tubular lumen predominantly in distal and much less in proximal tubular epithelial cells. However, it should be considered that the extent of toxicity does not necessarily reflect local intracellular concentrations, since also the cell type-specific susceptibility may be relevant. Interestingly, a second dose of 10 mg/kg b.w. OTA to the same mouse led to higher intracellular concentrations and a slower clearance in distal and to some degree also in proximal tubular epithelial cells. Although the mechanism explaining this phenomenon still has to be elucidated, this observation points to saturation of basolateral export mechanisms of the tubular epithelial cells after the second dose.

In the present study toxicokinetics of OTA in the liver is characterized by an increase of the OTA-associated signal in the blood of the sinusoids within seconds after injection, followed by uptake into hepatocytes and excretion into the bile canaliculi, where OTA is transiently enriched. From the canaliculi, small compounds have been shown to reach the interlobular bile ducts by a diffusion-dominated process (Vartak et al. 2021). It may appear surprising that the lower dose of 10 mg/kg b.w. OTA resulted in enrichment of the OTA-associated signal in the bile canaliculi while no increase in hepatocytes was detectable. This may be explained by an efficient OTA secretion by ATP-dependent carriers at the apical hepatocyte membrane from the cytoplasm into the canalicular space (Ghallab et al. 2019a; Kontaxi et al. 1996; Vartak et al. 2021) that prevents intracellular accumulation of the substrate at low concentrations. However, when the i.v. administered dose was increased to 20 mg/kg b.w. OTA, a clear intracellular increase was observed that may result from a saturation of canalicular secretion. This assumption is supported by the observation that the kinetics of the OTA-associated signal after injection of 20 mg/kg b.w. closely follows those of the concentration inside the hepatocytes. It is, however, remarkable that a much higher peak of the OTA signal appeared in the bile canalicular lumen than intracellularly in hepatocytes, which can also be directly observed in the corresponding videos. This transient canalicular enrichment may result from a situation where canalicular secretion by hepatocytes transiently exceeds the clearance capacity from canaliculi to bile ducts.

In both, kidney and liver, it appears critical to differentiate the early phase after i.v. injection of OTA and the steady-state afterward. In the early phase, when a higher fraction of non-protein bound OTA may be present, the free OTA may pass the glomerular barrier between blood and tubules, and similarly may be taken up from sinusoidal blood into hepatocytes to be excreted into canaliculi. However, in the steady-state where OTA is almost completely bound to plasma proteins, renal and hepatic excretion appears to be so slow that it cannot be detected in a single intravital imaging session of several hours.

A striking feature of the here observed AFB_1_ toxicokinetics is its rapid and complete clearance from sinusoidal blood with a half-life of approximately 4 min, which corresponds to the published short half-life in plasma of intravenously-injected mice (12.9 min; Wong and Hsieh 1980). With only a short time-lag, AFB_1_ enriched in hepatocytes and was secreted into bile canaliculi. An interesting feature is the AFB_1_-associated signal in the nuclei of hepatocytes that showed similar kinetics compared to the cytoplasm but reached higher peak intensities. In this respect, AFB_1_ differs from OTA that showed a stronger signal in the cytoplasm than in nuclei.

Major differences in toxicokinetics of AFB_1_ were observed in the pericentral and periportal lobular regions. In the cytoplasm of periportal hepatocytes, the AFB_1_-signal decreased rapidly with a half-life of ~ 9 min. However, in pericentral hepatocytes of the same lobules, the half-life was much longer (63 min). The difference may be explained by distinct metabolic activities in the different lobular zones. Cytochrome P450 isoforms (CYP3A11 and CYP3A13 in mouse) that metabolically activate AFB_1_ to reactive epoxides (Deng et al. 2018; Guengerich et al. 1998) show high activities in the pericentral but not in the periportal lobular region (Ghallab et al. 2019b). It has been reported that the AFB_1_–epoxides, in particular the exo-form, undergo hydrolysis to an 8,9–dihydrodiol product, which after rearrangement to a dialdehyde reacts with the lysine residues of proteins (Guengerich et al. 1998). The formation of AFB_1_ protein adducts may be an explanation why the AFB_1_-associated signal retained longer in the cytoplasm of pericentral than periportal hepatocytes. The peak of the AFB_1_-associated signal in nuclei was lower in the pericentral than in the periportal region. This may be because a high proportion of metabolically activated AFB_1_ in the pericentral lobular zone binds to cytoplasmic proteins so that less free compound diffuses into the nuclei. Interestingly, the nuclear half-life of the AFB_1_ signal was longer in the pericentral than in the periportal region (Table 3). This may be explained by the fraction of AFB_1_ epoxide formed in the cytoplasm of pericentral (but not in the periportal) hepatocytes that binds to DNA after diffusion into the nuclei.

Limitations of the present explorative study are that the mechanisms responsible for several of the here described partially unexpected observations remain to be analyzed. For example, the zonal clearance of AFB_1_ from the liver could further be analyzed by MALDI–MSI to study whether protein or DNA adducts are responsible for the delayed clearance. Also the unexpected finding that more OTA is taken up by the distal than the proximal tubular epithelial cells could be validated by the use of cell-specific reporter mice differentiating both proximal and distal tubular epithelial cells. It should also be considered that the intensity of the blue-fluorescent signal of OTA or AFB_1_ does not necessarily show a linear correlation with the tissue concentrations of these compounds. It has been shown that calibration of the fluorescent tissue signal with regard to tissue concentration of the analyte is possible, but the linear range is usually small (Jansen et al. 2017; Vartak et al. 2021). A further aspect to be considered is that rats are more susceptible to AFB_1_-induced hepatocarcinogenesis than mice (review: Hengstler et al. 1999). Thus, it will be interesting to compare the spatio-temporal kinetics in both species in future studies. In the present study, recording was done at a specific wavelength (780 nm) which shows maximum fluorescence emission for the parent OTA and AFB_1_; however, it should be considered that the formed metabolites/adducts may require a different excitation wavelength for fluorescence emission which was not addressed in the present study.

In conclusion, the present study shows that two-photon-based intravital imaging allows insights into spatio-temporal distribution of OTA and AFB_1_ in liver and kidney with subcellular resolution. This may provide valuable information in addition to conventional pharmacokinetic analysis.

## Supplementary Information

Below is the link to the electronic supplementary material.

Supplementary file1 (MP4 33389 KB)

Supplementary file2 (M4V 34378 KB)

Supplementary file3 (M4V 39686 KB)

Supplementary file4 (M4V 97514 KB)

Supplementary file5 (M4V 97578 KB)

Supplementary file6 (MP4 24409 KB)

Supplementary file7 (MP4 17335 KB)

## Abbreviations

AFB_1_: Aflatoxin B_1_
dTEC: Distal tubular epithelial cells
b.w.: Body weight
CLF: Cholyl–lysyl–fluorescein
i.v.: Intravenous
MALDI MSI: Matrix-assisted laser desorption/ionization mass spectrometry imaging
Min: Minute(s)
NTCP: Sodium taurocholate cotransporting polypeptide
OATPs: Organic anion transporting polypeptides
OTA: Ochratoxin A
p.o.: Per os
PBPK: Physiologically-based pharmacokinetic
TMRE: Tetramethylrhodamine–ethyl ester
